# Experimental Valorization of Recycled Palm Oil in Topical Formulations: Preparation, Characterization, and Antimicrobial Assessment

**DOI:** 10.3390/molecules31020335

**Published:** 2026-01-19

**Authors:** Paula Rusu, Andreea Creteanu, Alina-Mirela Ipate, Maricel Danu, Mirela-Fernanda Zaltariov, Daniela Rusu, Cristina Gabriela Tuchilus, Gladiola Tantaru, Gabriela Lisa

**Affiliations:** 1 Faculty of Chemical Engineering and Environmental Protection “Cristofor Simionescu”, Gheorghe Asachi Technical University of Iasi, 73 Prof.dr.doc D. Mangeron Street, 700050 Iasi, Romania; paula.simionescu@student.tuiasi.ro; 2 Faculty of Pharmacy, Grigore T. Popa University of Medicine and Pharmacy, 16 Universitatii Street, 700115 Iasi, Romania; acreteanu@gmail.com (A.C.); cristina.tuchilus@umfiasi.ro (C.G.T.); gtantaru2@yahoo.com (G.T.); 3 Petru Poni Institute of Macromolecular Chemistry, 41A Aleea Gr. Ghica Voda, 700487 Iasi, Romania; ipate.alina@icmpp.ro (A.-M.I.); zaltariov.mirela@icmpp.ro (M.-F.Z.); rusu.daniela@icmpp.ro (D.R.)

**Keywords:** recycled palm oil, semisolid cosmetic creams, rheology, microstructure, ATR-FTIR, SEM, DSC, contact angle, antimicrobial activity

## Abstract

Sustainable strategies for revalorizing food industry by-products are increasingly relevant in the development of modern experimental dermato-cosmetic formulations. In this study, two semisolid cosmetic creams (R10 and EM-R10) were designed using recycled palm oil—physically purified after intensive frying—as the lipid phase. The recycled oil was incorporated strictly within a controlled experimental framework and does not imply cosmetic-grade regulatory compliance. The formulations incorporated distinct bioactive profiles: R10 combined apricot and pineapple extracts with lime essential oil, while EM-R10 integrated fir bud and green tea extracts alongside the same essential oil. Both preparations contained Fragard as a preservative and niacinamide and panthenol as vitaminic components. The physicochemical properties of the formulations were assessed through rheology, confocal microscopy, ATR-FTIR, SEM, DSC, and contact angle measurements. Antimicrobial activity was evaluated against *Staphylococcus aureus*, *Escherichia coli*, *Pseudomonas aeruginosa*, and *Candida albicans* using disk diffusion and broth microdilution assays. The results demonstrate that, despite partial thermal degradation, recycled palm oil retains modified structural features that influence formulation-related properties relevant to topical systems. EM-R10 showed superior spreadability, adhesion, stability, and diffusion-related performance, as well as improved antimicrobial activity, within the investigated experimental conditions, highlighting recycled palm oil as a promising sustainable lipid phase for experimental dermato-cosmetic formulations, pending further purification, toxicological evaluation, and regulatory compliance assessment.

## 1. Introduction

The skin, accounting for approximately 15% of total body weight [1], is the body’s largest organ and serves essential protective, thermoregulatory, sensory, and vitamin D–synthesizing functions [2]. Beyond its physiological roles, skin health contributes significantly to overall well-being and self-perception, influencing both mental and physical status [3]. Skin frailty is associated with age, limited mobility, specific medical conditions, and chronic disease [4]. Although generally resilient, the skin’s integrity may be compromised by advanced age, cognitive impairment, dehydration, malnutrition, obesity, certain medications (including immunosuppressants, anti-inflammatory agents, and anticoagulants), incontinence, chronic or critical illness, impaired mobility, circulatory deficits, and radiation therapy [5].

As the primary interface between the body and the external environment, the skin is continuously exposed to physical, chemical, and biological stressors [6,7]. Consequently, topical cosmetic products play a key role in supporting skin barrier function, maintaining hydration, and improving sensory comfort [8].

In cosmetic science, creams and ointments are defined as semi-solid topical preparations intended to protect, moisturize, or condition the skin, without exerting pharmacological activity. Their performance depends strongly on the composition of the formulation base, which governs spreadability, occlusivity, sensory attributes, and the release of functional ingredients [9,10].

From a cosmetic perspective, protective formulations are designed to limit transepidermal water loss and shield the skin from external aggressors, while emollient products aim to improve skin softness and flexibility without implying therapeutic effects.

In recent years, increasing environmental concerns have encouraged the cosmetic industry to adopt more sustainable practices, including the use of renewable raw materials and the valorization of waste streams. Within this context, recycled vegetable oils have emerged as potential cosmetic ingredients, provided that their safety, purity, and physicochemical quality comply with applicable cosmetic regulations.

Palm oil, widely used in food and industrial applications, represents a significant waste resource after frying processes. However, oil subjected to intensive thermal treatment may contain oxidation products, polymerized lipids, and other degradation compounds, which require appropriate purification, stabilization, and quality control prior to cosmetic use [10].

In the present study, recycled palm oil was recovered after intensive frying and subsequently filtered and stabilized before incorporation into cosmetic formulations. The focus is placed on its technological and sensorial role as a lipid phase component, contributing to emolliency, texture, and occlusive properties, rather than on any pharmacological or therapeutic activity.

Shea butter (Vitellaria paradoxa) is a well-established cosmetic ingredient recognized for its emollient and skin-conditioning properties. It consists mainly of triglycerides rich in stearic and oleic acids, along with a minor unsaponifiable fraction containing triterpenes, tocopherols, phenolic compounds, and sterols [11]. These constituents contribute to antioxidant activity and skin comfort, supporting its use in cosmetic formulations intended to improve skin softness and barrier function, without implying medicinal action.

The emulsifying system employed in this work is based on an olive-derived emulsifying wax (INCI: cetearyl olivate and sorbitan olivate), a non-ionic oil-in-water emulsifier widely used in cosmetic formulations. This system provides good stability, pleasant sensory properties, and biodegradability, and functions as a structuring and emulsifying agent within the heated oil phase of the formulation.

Fir buds (*Abies* spp.) are a natural source of bioactive compounds, including phenolic compounds, flavonoids, and terpenes, which have been reported in the scientific literature to exhibit antioxidant and antimicrobial potential [12]. These properties may contribute to the oxidative stability of cosmetic formulations and to skin comfort, depending on the extraction method and concentration of active compounds. In this study, fir buds were incorporated by maceration in the lipid phase, aiming to enhance the functional and sensory profile of the cosmetic cream rather than to exert therapeutic effects.

This paper aims to investigate the feasibility of using recycled palm oil as a sustainable lipid phase within an experimental dermato-cosmetic context in cream formulations. The study evaluates the physicochemical characteristics, stability, and antimicrobial performance of the developed products, with emphasis on formulation quality, preliminary safety-related considerations, and formulation-relevant properties associated with skin feel and conditioning, under controlled laboratory conditions and without implying regulatory acceptance as a cosmetic-grade ingredient.

## 2. Results

### 2.1. Preparation Method for a Cream Containing Recycled Palm Oil (R10 and EM-R10)

Palm oil was collected from the student canteen located on the Tudor Vladimirescu campus in Iasi, affiliated with the “Gheorghe Asachi” Technical University, and recycled for the formulation of semisolid cream-type preparations for topical application on the skin. Selected simple substances were specifically adapted to the lipophilic nature of the type of oil being analyzed.

Thus, for the R10 cream, the following formula was used (Table A1).

The co-emulsifier used in the preparation of the R10 cream was an olive-based emulsifying wax (sorbitan olivate), which, when mixed with the recycled palm oil, increases the viscosity of the oily phase. The wax was prepared during the first step (Phase A), where it was melted together with the other lipid components in a water bath at 70 °C. Compared to other types of waxes, it reduces the greasy sensation and provides a pleasant, silky skin feel. Olive emulsifying wax ensures optimal dispersion of pigments and UV filters and improves the water resistance of creams.

The second preparation step included Phase B, in which two aromatic extracts were used: aromatic apricot extract and aromatic pineapple extract. The aromatic apricot extract is a water-soluble ingredient primarily used as a fragrance in cosmetic products. The pineapple extract, containing 53% (*w*/*w*) alcohol, plays an essential role in flavoring, imparting a characteristic pineapple scent to the product.

In the final step, only one type of oil was used in the form of lime essential oil (Citrus aurantifolia oil), which has multiple beneficial properties for the skin and the body. It is used in cosmetics for the care of oily and acne-prone skin and also acts as an effective antiseptic by stimulating the lymphatic system [13].

The preservative used was Fragard (also known as Naticide), a mixture of fractions of essential oils, aromatic compounds, and plant extracts with antifungal and antibacterial properties. It is a clear, low-viscosity liquid, with a density of 1.1 g/cm^3^ and a mild vanilla/almond scent. It was added in Phase C at a proportion of 0.96% (*w*/*w*), allowing uniform dispersion throughout the cream, and is effective within a pH range of 4.0–6.0. The R10 formulation exhibited a pH value of 4.5, measured immediately after preparation and again after one week, indicating good pH stability.

Niacinamide (Vitamin B3) is a beneficial ingredient for problematic skin, including acne-prone skin and rosacea-associated redness and inflammation. It has also been shown to be highly effective in cases of post-inflammatory hyperpigmentation and is well tolerated by all skin types [14].

Panthenol, used in this formulation in powder form, is the precursor of Vitamin B5, which, once it penetrates the skin, is rapidly converted into pantothenic acid, an essential component of cell and tissue activity. When applied to the skin in creams containing panthenol, it stimulates the synthesis of keratinocytes and promotes the proliferation of dermal fibroblasts, thereby supporting wound healing and accelerating skin regeneration. Additionally, it enhances skin hydration and exhibits anti-inflammatory effects [15].

The preparation of the R10 cream involved the separate heating of Phases A and B in a water bath until a temperature of 70 °C was reached. After removing the heat source and allowing a stabilization period of approximately five minutes, the contents of the beaker containing Phase A were slowly poured into the beaker containing Phase B. The mixture was then placed in a vessel with cold water and homogenized for three minutes using an electric mixer operating at 10,000 rpm (±15%). In the final stage, Phase C was incorporated after confirming that the temperature of the emulsion had dropped below 40 °C, under continuous mixing at the same speed for an additional three minutes. The resulting cream was labeled and transferred into brown glass containers for storage and further analysis.

For the EM-R10 cream, the following formula was used (Table A2). The first preparation stage (Phase A) included the fir bud extract obtained in the laboratory, first-press Non-GMO soybean oil with a saturated fatty acid content of 15.3 mL 19%, *w*/*w* and carbohydrates of 0.2 mL, 8%, *w*/*w*, and BIO shea butter, which is rich in phytosterols, polyphenols, and essential fatty acids and olive-based emulsifying wax (sorbitan olivate). The second preparation stage encompasses Phase B, in which a single extract was used, namely green tea extract, which has anti-inflammatory, skin-toning, antioxidant, anti-aging, anti-wrinkle, astringent, protective, and soothing properties. Olive wax, based on olive oil, was also used as a natural emulsifier. In the final stage, a single type of oil was used, specifically lime essential oil, alongside the preservative and regenerative elements. The preservative used was Fragard, and the vitamins and provitamins included were niacinamide and panthenol. For the EM-R10 type of cream, the same preparation method as for R-10 was followed. The recycled palm oil was replaced with fir bud extract in recycled palm oil. This extract was obtained in the laboratory through maceration, a process in which the plant material was ground using a mortar. Then, 25 g of the material was transferred into a dark-colored glass container, to which 100 mL of recycled palm oil was added. The container was kept in the dark at room temperature for 33 days. During the first three days and the last three days of the 33-day period, the container was placed on a heating plate at 50 °C for 30 min each day.

### 2.2. Physicochemical Evaluation of Fresh and Recycled Palm Oil

In the context of this study, recycled palm oil was incorporated into topical formulations strictly for experimental and research purposes. The formulations were designed to evaluate physicochemical behavior, structural organization, and antimicrobial performance, and do not imply regulatory acceptance of recycled oil as a cosmetic-grade ingredient under Regulation (EC) No 1223/2009.

The physicochemical properties of the filtered oil were analyzed and compared with those of unused palm oil. It was observed that during the frying process, the saponification index decreased relative to the initial oil, while the free acidity, acid value, and peroxide value increased (Table 1).

The comparative analysis of the physico-chemical properties of fresh and recycled palm oil highlights significant changes resulting from exposure to high temperatures and contact with atmospheric oxygen and food residues. While these transformations are typically associated with degradation from a nutritional standpoint, several studies indicate that thermally altered vegetable oils, following appropriate purification or refinement steps, may still be suitable as raw materials for non-food applications. In particular, oxidized lipid fractions and increased viscosity may be advantageous in cosmetic formulations, where emollient properties, texture modification, and occlusive effects are required, provided that safety and quality standards are met [16,17].

The slight decrease in density (from 904.06 to 902.02 kg/m^3^) and refractive index (from 1.4586 to 1.4578) suggests structural alterations in lipid compounds, likely due to oxidation of existing unsaturated bonds in fatty acids. Similarly, the observed reduction in kinematic viscosity may be attributed to the thermal breakdown of triglycerides into lower molecular weight compounds, a characteristic that can enhance skin absorption in topical formulations.

A notable decrease in surface tension (from 29.520 to 26.598 mN/m at 48 °C) indicates the presence of polar compounds at the oil interface, a feature of great relevance in emulsion systems where interfacial behavior directly impacts the stability of oil–water phases.

Changes in color (from 1.53 to 2.64) and the significant increase in peroxide value (from 3.80 to 13.00) confirm the progression of lipid oxidation. Lipid oxidation products are generally pro-oxidative, potentially irritating, and may be toxic; their potential application in cosmetics requires thorough safety and stability assessments, which are beyond the scope of this study. The use of recycled palm oil may be considered only under strictly controlled formulation conditions, combined with oxidation-stable oils and antioxidant extracts, and is limited to body care products, excluding facial or dermatological applications. The free acidity and acid value of recycled palm oil were higher than those of fresh palm oil, as expected due to the formation of free fatty acids during thermal processing and repeated use in frying.

The saponification value, although slightly reduced (from 186.29 to 183.89), remains within an acceptable range for the oil to serve as a viable source of fatty acids in soap synthesis or as a base for emulsifying agents.

### 2.3. Evaluation of Rheological Behavior

It is an established phenomenon in the field of cosmetic science that all cosmetic products exhibit flow behavior, even if this flow is not always perceptible to the consumer [18]. Each category of cosmetic products possesses specific rheological characteristics [19]. The manufacturer of a cream product must consider two factors: the flow behavior of the materials and the conditions that should prevent flow.

**Figure 1:**
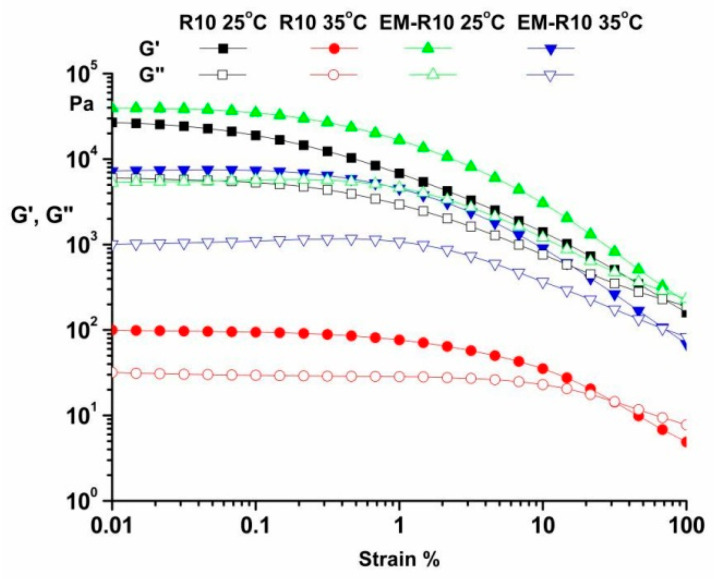
Amplitude sweep for R10 and EM-R10 samples at 25 °C and 35 °C.

The amplitude sweep, ranging from 0.01 to 100%, was conducted at temperatures of 25 °C and 35 °C, with a frequency of 10 rad/s. The results for the R10 and EM-R10 samples are presented in Figure 1. These experiments enabled the determination of the linear viscoelastic region (LVR), which corresponds to dynamic moduli that are independent of strain. As illustrated in Figure 1, both samples demonstrated a predominant elastic behavior (G′ > G″) within the LVR for both temperatures, indicating their solid viscoelastic character and good physical stability under quiescent conditions [20].

The limit of the LVR was determined as the point where the storage modulus G′ plateau value dropped (γLVR = 0.01% for both samples) [15,21].

**Figure 2:**
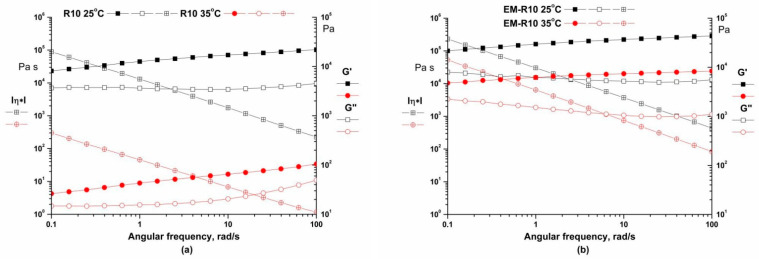
Frequency sweep for R10 (**a**) and EM-R10 (**b**) samples at 25 °C and 35 °C.

Frequency sweeps ranging from 0.01 to 100 rad/s were carried out at temperatures of 25 °C and 35 °C, with a constant strain in the LVR of 0.01%. This test is designed to demonstrate the short-term and long-term physical stability of the samples. As illustrated in Figure 2, the storage modulus and loss modulus are found to be weak functions of frequency, with G′ > G″, indicating that the physical network structure has been built up and exhibits solid-like behavior. The viscoelastic properties of the material are attributable to the interactions among its components, which include cross-linking, entanglement, and aggregation. The structural changes observed in both samples were found to be consistent over time, suggesting that the structural integrity of the samples was maintained during rest periods [20,22]. This indicates that the creams exhibited adequate shelf-life stability [23].

The samples exhibited predominantly elastic behavior across a range of frequencies, suggesting that the materials possess good storage stability. It is hypothesized that the dynamic moduli exhibit a weak dependency on frequency, which suggests that the samples are more amenable to storage due to their network firmness, which is predicated on a cross-linked structure and strong bonding [24].

**Figure 3:**
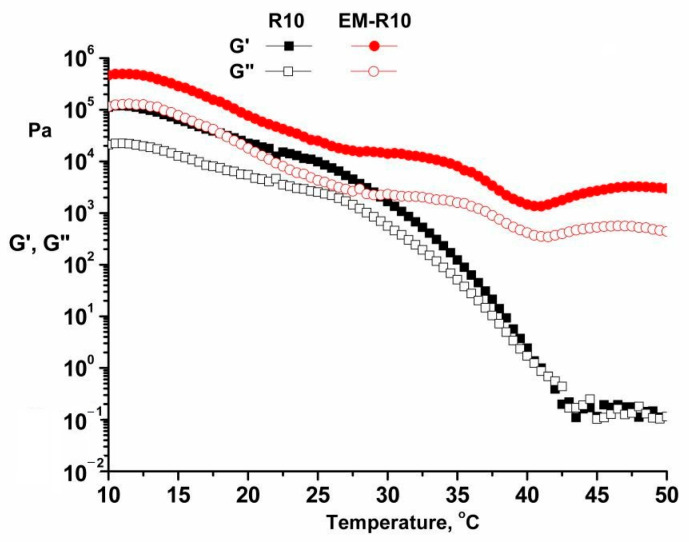
Temperature sweep test for R10 and EM-R10 samples.

The temperature sweep test, conducted within the range of 10–50 °C at a heating rate of 0.5 °C/min, employed a constant frequency (1 Hz) and constant strain (within the LVR of 0.01%), as depicted in Figure 3 for the R10 and EM-R10 samples.

As the temperature is increased, the internal structure does not maintain its original configuration. This results in a decrease in the dynamic moduli as the temperature increases. The storage modulus exhibits a greater magnitude than the loss modulus across a range of temperatures. As the temperature rises, each ingredient begins to melt, leading to the destruction of the network structure. It is anticipated that creams will maintain their intrinsic characteristics of thickness at skin temperature (35 °C).

As evidenced by the data, there is a relationship between the storage modulus and loss modulus of the R10 sample. The findings indicate a decrease in the difference between these moduli, suggesting a transition from a non-Newtonian to a Newtonian fluid behavior as the temperature increases.

It has been demonstrated that the distribution of the sample would be more efficient if it were applied in a thin layer to the skin at 35 °C, in comparison to the EM-R10 sample [24].

**Figure 4:**
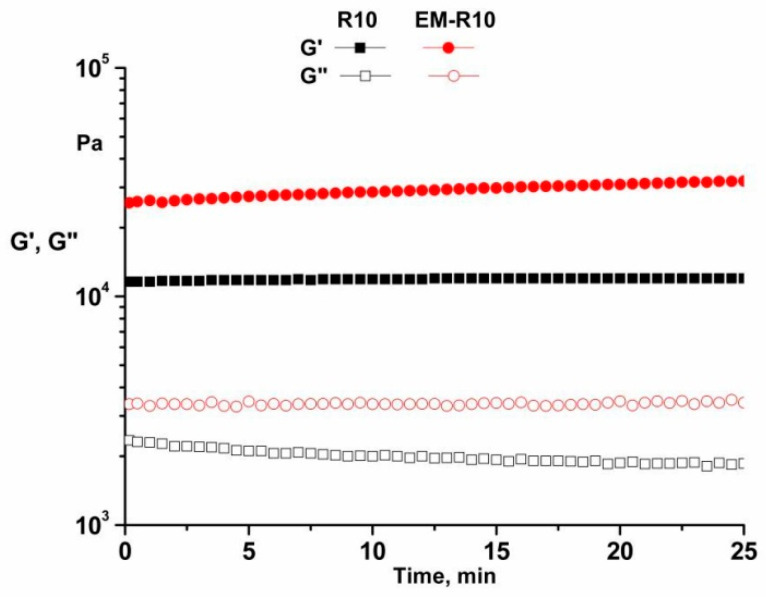
Oscillatory time test for R10 and EM-R10 samples at 25 °C.

The oscillatory time test was performed at a temperature of 25 °C, with a constant strain of 0.01% (in the LVR) and a constant frequency of 1 Hz. The results obtained for R10 and EM-R10 samples (Figure 4) demonstrate that both the G′ and G″ moduli remain constant for 25 min. It is evident that the analyzed samples exhibit adequate time stability.

**Figure 5:**
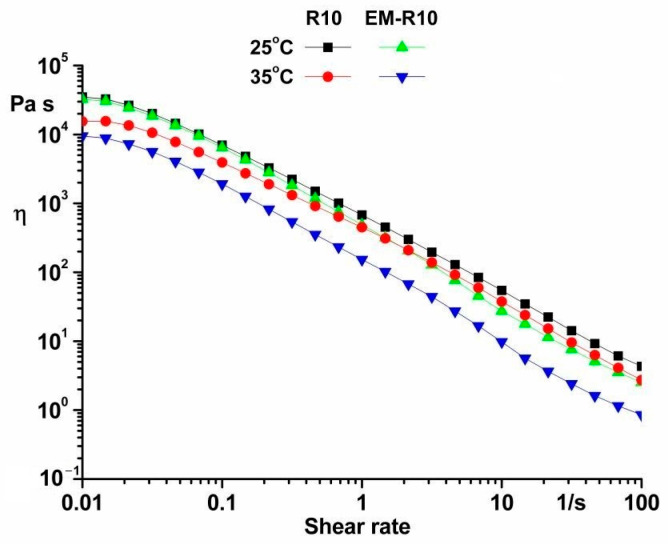
Viscosity curves (η vs. shear rate) for R10 and EM-R10 samples at 25 °C and 35 °C.

Viscosity curves ranging from 0.01 to 100 s^−1^ were conducted at temperatures of 25 °C and 35 °C. The rheological tests of the R10 and EM-R10 samples enabled an investigation of viscosity variation as a function of shear rate (Figure 5).

The characterization of both samples included the observation of shear-thinning behavior, suggesting a yield stress. It has been determined that this behavior is considered desirable for most topically formulated products. The advantages of this behavior include facilitating the spreading of the product on the skin and the removal of the product from the container. The variations in the viscosity values of the samples can be attributed to compositional differences and the temperatures at which they were tested [23].

It has been observed that the ease with which a product is distributed on the skin is directly proportional to the magnitude of its yield stress. This finding suggests that yield stress is a key factor in determining the thickness of the product film layer on the skin’s surface [24,25].

The Newtonian flow region is observed at low shear rates, and the viscosity of the samples is significantly reduced as the shear rate increases, indicating that both products exhibit shear-thinning behavior.

The observed shear-thinning behavior in the samples can be attributed to modifications in their microstructure. At low shear rates, the samples exhibit high viscosity, attributable to their network structure. An increase in shear rate has been shown to result in a decrease in the number of entanglements between components. This decrease in entanglements leads to the destruction of the structure and a decrease in viscosity [24,26].

**Figure 6:**
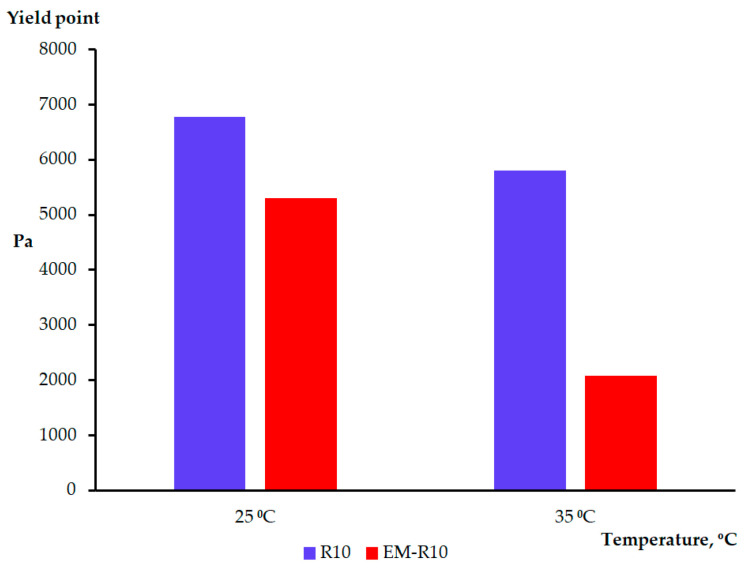
The yield stress for samples at 25 °C and 35 °C.

The samples could be fitted by the Herschel–Bulkley model (y = a + b·x^p^; a—yield stress; b—viscosity; p—regression parameters; x—shear rate; y—shear stress) in order to determine their yield stress (a) (Figure 6). The values of the parameters a, b, p, and the correlation coefficient R^2^ are presented in Table A3.

It has been posited that the samples exhibited high yield stress values, indicative of inherent resistance to flow, attributable to their network structure [14]. The values of the yield stress serve as an important indicator of the consistency of a cosmetic product.

The values of viscosity at 0.1, 1, 10, and 100 s^−1^ were recorded for each sample at 25 °C (Table 2). The various corresponding shear rate ranges can be associated with physical operations commonly employed in the context of creams. These operations include draining under gravity at rates ranging from 0.01 to 10 s^−1^, as well as creaming, spooning, and pouring at rates between 10 and 100 s^−1^ [21,27].

It is evident for both samples, R10 and EM-R10, within the shear rate range of 0.01 to 0.1 s^−1^, a range characteristic of gravity forces and very low vibrations, that the viscosity values are high. This finding suggests that the stability of the samples is maintained, with no separation of their components over time [28].

### 2.4. Study of Phase Behavior of Creams via Confocal Microscopy

To analyze the morphology and microstructural stability of the R10 cream.

**Figure 7:**
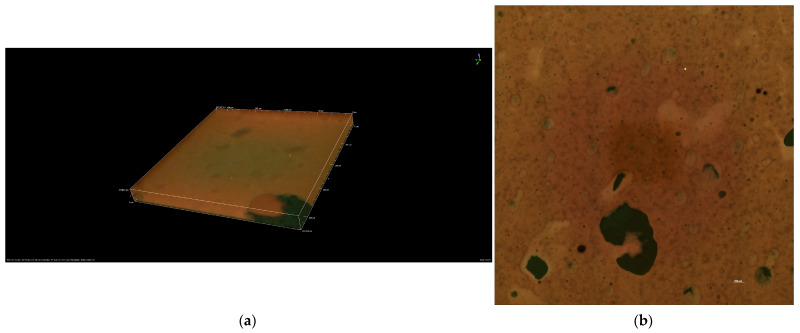
Images obtained through confocal microscopy for R10 cream: (**a**) 3D reconstructed confocal image (volume rendering) of the R10 cream sample; (**b**) 2D confocal micrograph (XY optical section) of the R10 cream.

The results presented in Figure 7a,b highlight the stability of the R10 cream. Figure 7a is a 3D representation at dimensions of 441.94 μm × 441.94 μm × 37.98 μm. The measured values on the X and Y axes are 0.22 μm, and on the Z axis, 1.81 μm. The image was captured at 100 pixels.

The confocal image shows a fine network of spherical structures, typical of this cream. The aqueous droplets are well-dispersed and show no significant tendency to coalesce. The aqueous phase globules exhibit a relatively homogeneous size, suggesting good emulsification due to the presence of olive wax emulsifier.

The results obtained and presented in Figure 7b for R10 cream were captured using a CFI Plan Apochromat Lambda D 10X objective at a wavelength of 50 μm. The R10 cream is well stabilized and shows no visible phase segregation. The absence of large aggregates suggests that the cream is stable in the short term.

### 2.5. Characterization with ATR-FTIR Technique

**Figure 8:**
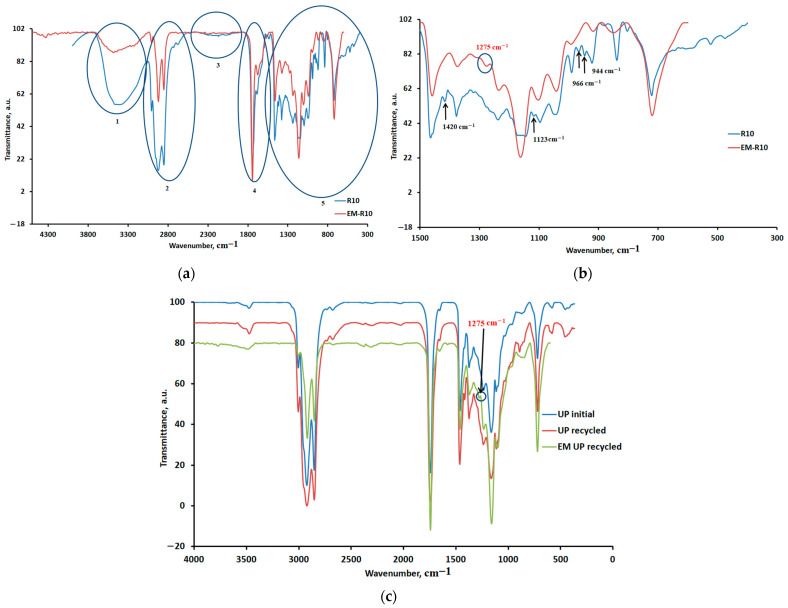
(**a**) ATR-FTIR spectra for R10 and EM-R10 creams; (**b**) ATR-FTIR spectra for region 5 (R10 and EM-R10 creams); (**c**) ATR-FTIR spectra for initial palm oil (UP initial), recycled palm oil (UP recycled), and EM recycled palm oil (EM UP recycled).

In this study, ATR-FTIR spectra were recorded for the R10 cream containing recycled palm oil and the EM-R10 cream containing fir bud extract in recycled palm oil. The results obtained are presented comparatively in Figure 8a. According to the spectra shown in Figure 8a, the most significant changes in the ATR-FTIR spectrum for the EM-R10 cream compared to R10 occur in regions 1, 2, and 5, where the peaks are more intense for the R10 cream compared to the EM-R10 cream. Figure 8b presents the ATR-FTIR spectra for region 5 (R10 and EM-R10 creams), and Figure 8c presents the ATR-FTIR spectra for initial palm oil (UP), recycled palm oil (UP recycled), and EM recycled palm oil (recycled palm oil in which fir buds were macerated).

The peak in the 3008/3006 region (Figure 8a) is significantly reduced, indicating a lower amount of unsaturated acids in the EM-R10 cream compared to R10. The food frying process in palm oil has a significant effect on the degree of unsaturation [29]. According to the ATR-FTIR spectra in Figure 8b, the appearance of a new peak at 1275 cm^−1^ in the EM-R10 cream is also evident, which can be associated with the vibration of the –C-O-C– bonds specific to ether groups [30,31]. This peak is also present in the ATR-FTIR spectrum of the recycled palm oil in which fir buds were macerated (EM UP recycled), as shown in Figure 8c. In Figure 8b, the disappearance of peaks associated with aliphatic and ester groups is also highlighted.

### 2.6. Determination of Contact Angle

The experimental determination of the contact angle for R10 and EM-R10 creams.

The results obtained are presented comparatively in Table 3 for water and Table 4 for ethylene glycol. For the R10 cream, four measurements were made for water and three for ethylene glycol. For the EM-R10 cream, four measurements were made for both water and ethylene glycol.

For the EM-R10 cream, a decrease in the contact angle was observed compared to the R10 cream. Additionally, the work of adhesion nearly doubled, and the solid–liquid interfacial tension decreased significantly for EM-R10 compared to R10.

The results from the contact angle analysis show a significant difference between the two formulations. The EM-R10 cream, which contains fir bud extract in recycled palm oil, has a lower contact angle than the R10 cream, indicating better wetting of the glass surface. This behavior suggests a more favorable interaction between the liquid phase and the solid substrate, which can be attributed to the bioactive compounds in fir buds (such as resin acids, flavonoids, and terpenes), which may act as natural surfactants and enhance the local polarity of the system. Furthermore, the work of adhesion value for EM-R10 is approximately doubled, supporting the idea of stronger intermolecular interactions at the solid–liquid interface. The reduction in solid–liquid interfacial tension indicates increased system stability when in contact with surfaces, which is consistent with its composition, especially the presence of green tea extract, which is rich in polyphenols that can interact effectively with polar surfaces. In conclusion, plant extracts with a polar or amphiphilic chemical profile positively influence the interfacial behavior of creams, improving adhesion and compatibility with solid surfaces.

The results presented in Table 4 indicate that for ethylene glycol, there are no significant changes in the static contact angle (θ) and work of adhesion (Wa). However, the solid–liquid interfacial tension (γsl) decreases from 30 mN/m for R10 to 21 mN/m for EM-R10, which can be associated with a redistribution of surface tensions and a better adaptation to contact with polar liquids such as ethylene glycol. Thus, although the contact angle with ethylene glycol does not change substantially, the total interfacial energy is reduced, supporting the concept of effective adhesion.

In conclusion, plant extracts rich in polar and amphiphilic compounds not only enhance the bioactive potential of the cream but also significantly influence its interfacial behavior, contributing to a more stable and effective formulation in contact with various surfaces.

### 2.7. Characterization Using the SEM Technique

**Figure 9:**
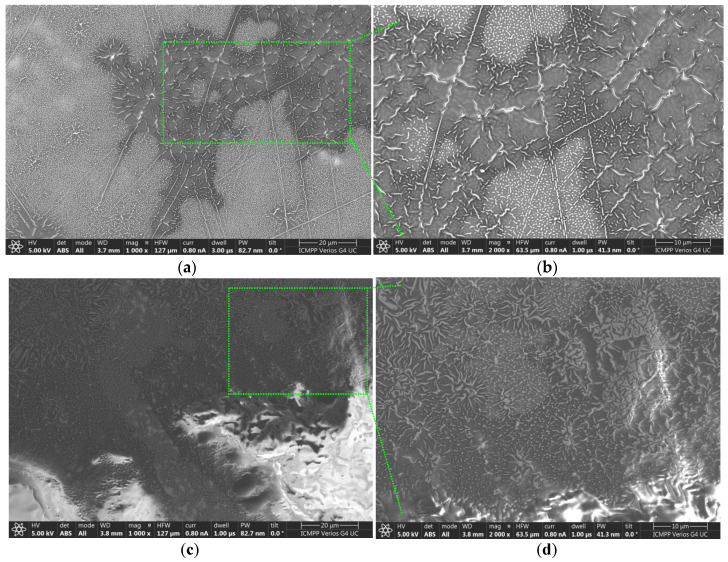
SEM images of the Strat-M^®^ membrane on which the R10 cream was applied: (**a**,**c**) at magnifications of 1000×; (**b**,**d**) at magnifications of 1000×.

**Figure 10:**
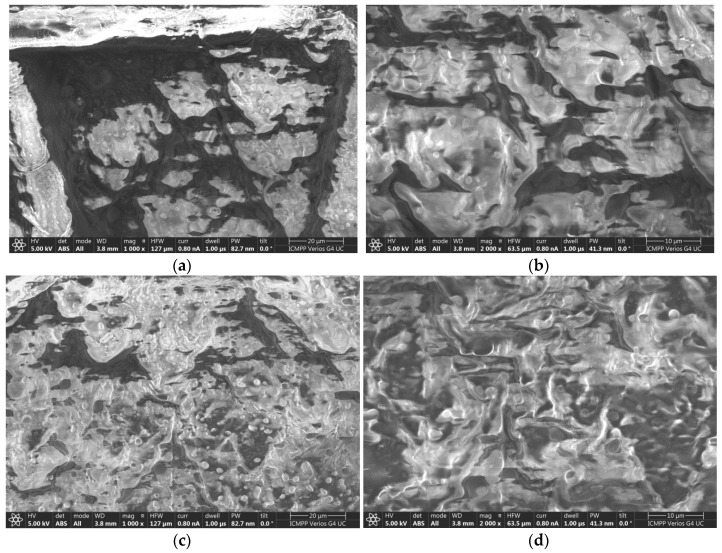
SEM images of the Strat-M^®^ membrane on which the EM-R10 cream was applied: (**a**,**c**) at magnifications of 1000×; (**b**,**d**) at magnifications of 1000×.

The morphology of the membrane surfaces to which the aforementioned creams R10 and EM-R10 were applied was assessed at magnifications of 1000× and 2000×. The results obtained are presented in Figure 9 for the R10 cream and in Figure 10 for the EM-R10 cream.

The analysis of the images presented in Figure 9 highlights the structure of the Strat-M^®^ membrane, which mimics the epidermis of human skin. In the folds on the surface of the membrane (Figure 9b,d), films of the R10 cream are visible, protecting the membrane and providing it with a hydrophobic character, as evidenced by the contact angle value measured for this sample (95°).

In the case of the EM-R10 cream, which contains palm oil in which fir buds were macerated, the SEM images in Figure 10a,c highlight the presence of droplets that are uniformly distributed over the cream film applied to the synthetic membrane, as well as partially covered areas where pores are present. This cream exhibits a hydrophilic character, as indicated by the measured wetting angle (54°), which was presented in the previous subsection. The hydrophilic nature of this cream will allow for deeper penetration into the layers of the skin structure [32].

### 2.8. DSC Analysis of Melting and Crystallization Behavior of Palm Oil-Based Creams

**Figure 11:**
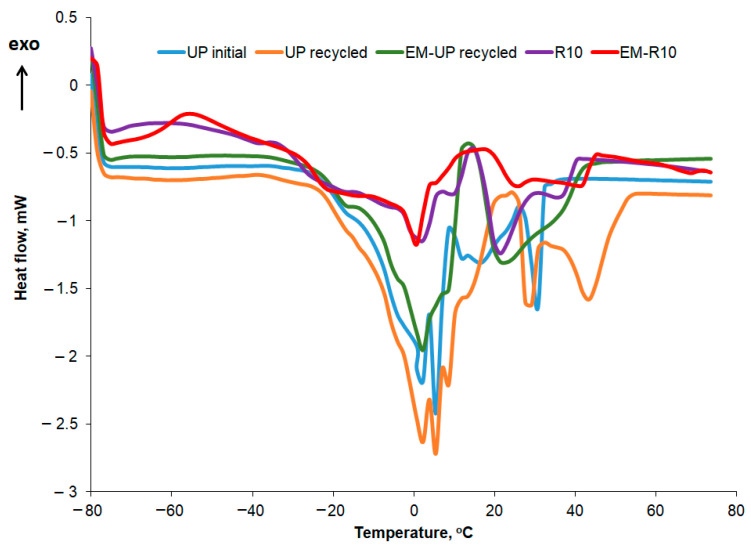
DSC curves for samples in the second heating stage.

The DSC curves recorded for the second heating stage at a rate of 5 °C/min are presented comparatively in Figure 11 for the R10 cream, EM-R10, UP initial, UP recycled, and EM-UP recycled.

In Table 5, the temperatures at which the melting peaks occur are presented, while the corresponding melting enthalpy values for each peak are shown in Table 6. In the case of palm oil, the polycrystallinity is more pronounced, with several melting peaks distinguishable, the most intense ones occurring at 16.63 and 30.45 °C for UP initial, and at 2.30, 5.05, 6.65, 8.72, 15.06, 28.31, 43.48 °C for UP recycled. The variation profile of the heat flow with increasing temperature is similar to what other authors have reported for palm oil in the literature [33]. The peak at 43.48 °C in the DSC heating curve of the recycled palm oil sample can be associated, according to the literature [34], with the presence of animal fats. The melting enthalpy values for recycled palm oil show slight changes compared to those obtained for the initial oil. For the first five peaks, the melting enthalpy value decreases, while in the higher temperature range (peaks 6, 8, and 9), the melting enthalpy (ΔH) increases.

**Figure 12:**
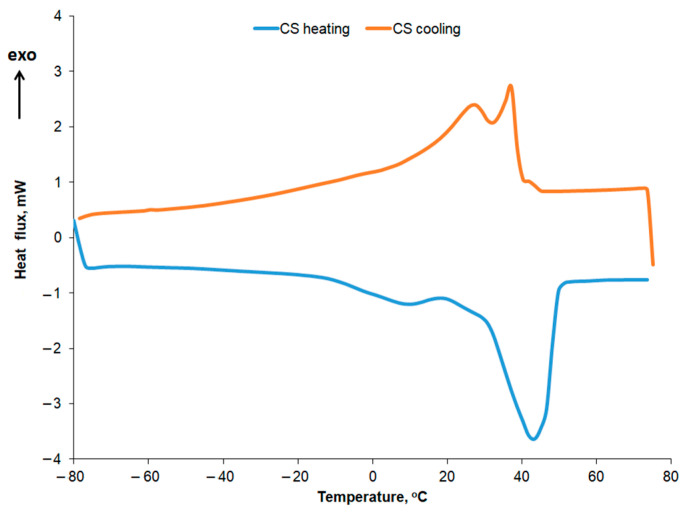
DSC curves for olive wax.

For the R10 and EM-R10 creams, the polycrystallinity is less pronounced, with some of the peaks identified in recycled palm oil also being observed. Olive wax was used as an emulsifier in the composition of the R10 and EM-R10 creams. The DSC melting and crystallization curves for olive wax (CM) are shown in Figure 12. Five melting and crystallization peaks are highlighted in the positive temperature range, typical for hydrogenated oils.

**Figure 13:**
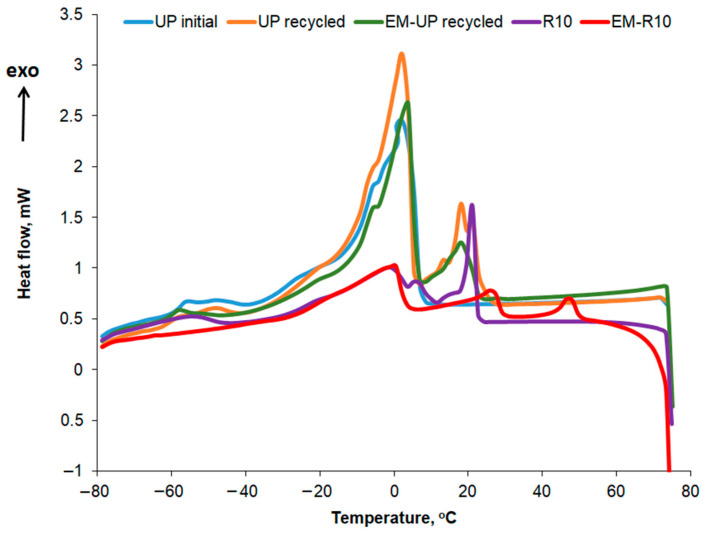
DSC cooling curves for UP initial, UP recycled, EM-UP recycled, R10, and EM-R10.

The crystallization curves for the creams using recycled palm oil are compared in Figure 13, while the main thermal characteristics and crystallization enthalpies are shown in Table 7 and Table 8. In the crystallization curves of both the fresh and recycled palm oil, a slightly more pronounced peak is observed at approximately 3 °C, along with several smaller peaks at the temperatures listed in Table 7. For the recycled palm oil, the crystallization curve also exhibits two more intense peaks at 18 °C and 21 °C, respectively. The peaks around 3 °C correspond to the crystallization of the oleic acid fraction, while the peaks at higher temperatures can be attributed to the crystallization of the stearic acid fraction [35]. Crystallization peaks at sub-zero temperatures, specifically at −40 °C, were also reported by Almeida et al. for two types of palm oil [36]. According to the data presented in Table 8, the crystallization enthalpy is approximately the same for both types of palm oil—initial and recycled—for the peak at around 3 °C. This similarity in enthalpy suggests that the recycling process does not significantly alter the low-melting triglyceride fraction responsible for the 3 °C peak. The observed crystallization behavior is consistent with previous studies reporting stable oleic acid fraction crystallization in palm oils [35,36], supporting the conclusion that recycled palm oil retains the same low-temperature crystallization characteristics as fresh palm oil.

In the case of the R10 and EM-R10 creams, several peaks observed in the crystallization curves of the recycled palm oil or the emulsifying agent (olive wax for both R10 and EM-R10) are also present. The highest crystallization enthalpy is recorded for the EM-R10 cream at the peak corresponding to approximately 10 °C.

### 2.9. Antimicrobial Susceptibility Tests

The diameters of the inhibition zones (in mm) corresponding to the tested compounds are shown in Table 9. Results are expressed as means ± SD (Standard Deviation).

The tested samples did not demonstrate antibacterial activity against Gram-negative bacteria. However, R10 and EM-R10 showed inhibitory activity against the Gram-positive reference strain *S. aureus* ATCC 25923.

A good antifungal activity was observed against *Candida albicans* for both tested samples (Table 9). The low SD values indicate good repeatability of the measurements.

The antimicrobial spectrum of these compounds may be related to differences in their qualitative and quantitative chemical composition.

## 3. Discussion

Thermal processing and recycling of palm oil induced measurable physicochemical changes, reflected by decreased density, refractive index, viscosity, and surface tension, together with increased peroxide value and color index. These modifications are indicative of lipid oxidation and partial triglyceride degradation, which alter interfacial behavior and may influence emulsion stability and skin interaction. While such changes can enhance spreadability, they also raise concerns regarding oxidative stability and safety, limiting the direct cosmetic use of recycled palm oil without formulation optimization.

Rheological analysis showed that both R10 and EM-R10 creams display solid-like viscoelastic behavior (G′ > G″) with minimal frequency dependence, indicating the presence of stable internal network structures. The persistence of this behavior up to skin temperature suggests adequate structural stability during application. The shear-thinning flow and measurable yield stress observed for both formulations are desirable characteristics for topical products, ensuring storage stability at low shear and good spreadability under application conditions.

Spectroscopic and microscopic analyses confirmed structural and compositional differences between the formulations. The reduced unsaturation level and the emergence of ether-related bands in the EM-R10 cream reflect chemical modifications induced by oil recycling and interactions with plant extracts. These molecular changes contribute to improved wetting, adhesion, and reduced solid–liquid interfacial tension, highlighting the role of polar and amphiphilic bioactive compounds in modifying interfacial properties.

Thermal analysis revealed reduced polycrystallinity in the cream formulations compared to recycled palm oil, due to the presence of olive wax emulsifier, which promotes a more homogeneous crystalline structure. The higher crystallization enthalpy observed for EM-R10 suggests stronger molecular interactions within the formulation. Additionally, both creams exhibited selective antimicrobial activity against Gram-positive bacteria and fungi, which may be associated with their compositional differences and bioactive content.

Overall, the findings indicate that recycled palm oil significantly influences the physicochemical, rheological, and interfacial properties of cosmetic creams. When combined with plant extracts, these effects may be functionally advantageous; however, careful formulation design and stability control are essential to ensure safety and performance.

## 4. Materials and Methods

**Figure A1:**
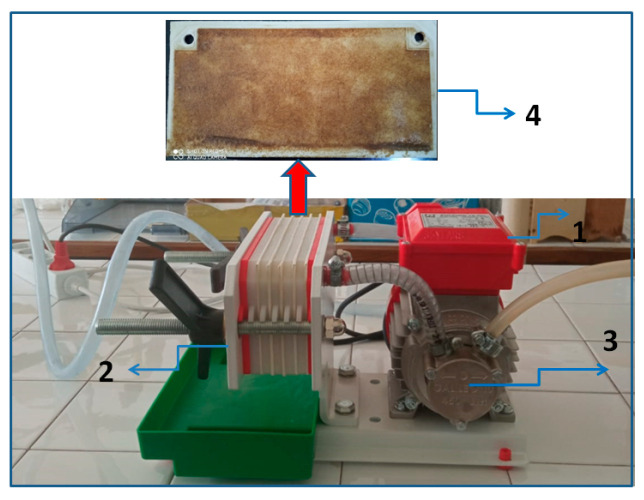
Filtration Installation Scheme. 1—1. electric pump; 2—multilayer compressed filtration system; 3—340 W electric motor; and 4—filter.

The recycled palm oil was obtained from used frying oil collected from a canteen and subjected to a physical recycling process (Figure A1). Prior to filtration, the oil was heated to 50 °C to reduce viscosity. Filtration was performed using a Rover PULCINO plate oil filter equipped with five cellulose filter plates (19 cm × 9 cm), providing a total filtration surface area of 0.171 m^2^. No chemical refining steps (e.g., neutralization, deodorization, bleaching, or adsorption treatments) were applied. In this study, the term recycled oil refers exclusively to physically filtered used frying oil, employed as a model lipid phase for experimental evaluation rather than as a cosmetic-grade raw material.

To obtain a plant-based extract, 25 g of fir buds were added to the recycled palm oil. The buds were crushed using a mortar, weighed, and transferred into a dark glass container, after which 100 mL of recycled palm oil was added. The mixture was subjected to a maceration process in the laboratory.

Two creams were subsequently prepared using both plain recycled palm oil and macerated oil containing fir buds. These formulations, designated R10 and EM-R10, respectively, were produced according to the procedures described in Section 2.

The semisolid preparations, intended for topical application, were characterized using the following analytical techniques: rheological analysis, confocal microscopy, attenuated total reflectance–Fourier transform infrared spectroscopy (ATR-FTIR), contact angle measurements, scanning electron microscopy (SEM), and differential scanning calorimetry (DSC). In addition, antimicrobial activity was assessed against Gram-positive bacteria (*Staphylococcus aureus*, ATCC 25923), Gram-negative bacteria (*Escherichia coli*, ATCC 25922; *Pseudomonas aeruginosa*, ATCC 27853), and a pathogenic yeast (*Candida albicans*, ATCC 10231). The antimicrobial activity was assessed using disk diffusion and broth microdilution techniques [37,38]. For the disk diffusion assay, Mueller–Hinton agar (Oxoid) and Mueller–Hinton Fungi agar (Biolab) were inoculated with suspensions of the tested microorganisms.

Sterile stainless-steel cylinders (5 mm internal diameter, 10 mm height) were placed on the agar surface in Petri plates. Subsequently, 100 µL of the tested compounds (R10) and (EM-R10), each at a concentration of 60 µg/mL, were dispensed into the cylinders. Plates were kept at room temperature for 10 min to allow uniform diffusion, then incubated at 35 °C for 24 h. Commercial disks containing Ciprofloxacin (5 µg/disk) and Fluconazole (25 µg/disk) served as reference antimicrobial agents. Following incubation, inhibition zone diameters were recorded. All antimicrobial assays were performed in triplicate (*n* = 3). The inhibition zone diameters were measured independently for each replicate, and results were expressed as mean ± standard deviation (SD). Due to the exploratory nature of the antimicrobial screening and the limited number of tested samples, no inferential statistical analysis was applied.

Rheological characterization of the creams was carried out using a Physica MCR 501 modular rheometer (Anton Paar, Graz, Austria), equipped with a Peltier temperature control system. The analyses included amplitude sweep and frequency sweep tests, temperature ramp tests, oscillatory time tests, and viscosity curve determinations. A parallel plate geometry was employed throughout the measurements. To check the reproducibility of the rheological results, three specimens of each sample were analyzed.

For the microscopic analysis of the creams, a Nikon AX confocal microscope (Nikon Instruments Inc., Tokyo, Japan) was employed. This model belongs to Nikon’s 10th-generation confocal microscope series. Laser scanning in confocal microscopy has become a highly effective tool for evaluating thin-section samples, with thicknesses up to 100 μm. In a confocal microscope, the image is generated by scanning the sample with one or more focused laser beams. The system offers a very high pixel density, enhanced sensitivity, fast scanning speed, and a large field of view of 25 mm. The AX/AX R confocal microscope systems capture an exceptional level of detail and incorporate a suite of artificial intelligence-based software tools that streamline confocal imaging workflows and simplify data extraction.

The Attenuated Total Reflection–Fourier Transform Infrared Spectroscopy (ATR-FTIR) technique was applied to the creams, the initial palm oil, the recycled palm oil, and the recycled palm oil macerated with fir buds. A Bruker Vertex 70 spectrometer (Bruker Optics, Ettlingen, Germany) was used for this analysis. The ATR spectra were recorded in the 4000–600 cm^−1^ range, with a resolution of 4 cm^−1^ and 32 scans per spectrum, at room temperature. The obtained spectra were processed using OPUS software (version 6.5, Bruker Optics), applying baseline corrections and normalization procedures to ensure accurate interpretation of the absorption bands.

The contact angle measurements of the semisolid formulations applied to glass slides were performed using a CAM-101 system (KSV Instruments, Helsinki, Finland). This equipment determines static contact angles of liquids by analyzing the drop shape. Water and ethylene glycol were used as solvents, and the measurements were carried out at 25 °C. A 1 mL Hamilton syringe (Hamilton Company, Reno, Nevada) was used to dispense the drops, which had volumes of approximately 1 μL for water and 1.6 μL for ethylene glycol. The contact angle was measured immediately after the drop was applied to the surface, using the KSV CAM software (version 4.02) for image analysis. The contact angle values reported in this study represent the average of 3 to 6 measurements per sample, with a standard deviation of less than 6%.

The prepared creams were applied onto Strat-M^®^ membranes (Merck, Darmstadt, Germany), allowed to dry, and subsequently evaluated using a Quanta 200 Scanning Electron Microscope (FEI Company, Hillsboro, OR, USA). Scanning Electron Microscopy (SEM) enabled the morphological assessment of the membranes onto which the semisolid formulations had been applied.

Differential Scanning Calorimetry (DSC) curves were recorded using a Mettler Toledo DSC1 instrument (Mettler Toledo, Greifensee, Switzerland) under an inert nitrogen atmosphere at a flow rate of 150 mL/min. The sample mass for analysis was approximately 5 mg, and a heating rate of 5 °C/min was applied over the temperature range of −80 °C to 75 °C, including two heating cycles and one cooling cycle. The mass of the analyzed samples ranged between 4.6 and 5.1 mg. The thermograms were processed using STAR^e^ Software version SW9.10 to identify the main thermal characteristics.

## 5. Conclusions

The cosmetic formulation developed in this study integrates bioactive compounds with complementary nutritional and cosmetic functions, commonly employed in advanced dermato-cosmetic formulations. The recycled palm oil, recovered after an intensive frying process and subsequently filtered, maintained its main physicochemical parameters (density, refractive index, and kinematic viscosity) while showing significant variations in surface tension, color index, and specific heat capacity. These changes may enhance the interfacial behavior of the final product.

The main components, shea butter, olive emulsifying wax, plant extracts (fir bud, green tea, apricot, and pineapple), lime essential oil, and a vitamin complex (Niacinamide and Panthenol) confer regenerative, antioxidant, antimicrobial, and emollient properties. Structural and physicochemical analyses (SEM, confocal microscopy, ATR-FTIR, and DSC) confirmed a well-stabilized morphology with no visible phase separation. Compared to R10, the EM-R10 cream exhibited a more hydrophilic interfacial characteristic, resulting in improved skin penetration and more efficient absorption of active ingredients.

Rheological characterization revealed a shear-thinning and predominantly elastic behavior (G′ > G″), indicating good spreadability and mechanical stability under storage conditions. The antimicrobial evaluation demonstrated notable inhibitory activity against *Staphylococcus aureus* and *Candida albicans*, confirming the synergistic effects of the phytochemical constituents and their potential for topical protection.

Overall, the findings highlight the feasibility of using thermally processed and recycled palm oil as an alternative raw material in sustainable cosmetic formulations. Although partial degradation occurs during frying, the resulting physicochemical modifications can be advantageous in optimizing cream performance. However, it should be emphasized that no experimental safety assays (e.g., cytotoxicity or skin irritation tests) were performed in this study. Therefore, while the physicochemical and functional characteristics are promising, the topical application of these formulations requires comprehensive safety and stability assessments prior to any human use.

Thus, the upcycling of lipid-rich waste materials represents a viable strategy for the development of stable, effective, and environmentally responsible dermato-cosmetic products, provided that rigorous safety evaluations are conducted before consumer application.

## 6. Limitations and Future Perspectives

The recycled palm oil used in this study was obtained under specific frying conditions, which may limit the broader applicability of the results. Long-term and accelerated stability tests, as well as comprehensive toxicological and dermatological evaluations, were not conducted and are required to fully assess formulation safety and shelf-life. In addition, antimicrobial activity was evaluated against a limited number of reference strains. Future studies should focus on standardized recycling protocols, incorporation of antioxidants and oxidation-stable oils, extended stability testing, and advanced skin interaction assessments to support the safe and sustainable use of recycled palm oil in cosmetic formulations.

## Figures and Tables

**Table 1:** The physico-chemical properties of fresh and recycled palm oil.

Property	Palm Oil	Recycled Palm Oil	Temperature, °C
Denisty ρ (kg/m^3^)	904.06 ± 0.96	902.02 ± 1.12	50
Refractive Index	1.4586 ± 0.0002	1.4578 ± 0.0001	50
Kinematic Viscosity (mm^2^/s)	34.86 ± 0.01	34.48 ± 0.10	
Surface Tension γ (mN/m)	^a^ 29.520 ± 0.049	^b^ 26.598 ± 0.651	^a^ 47.759 ± 2.114 ^b^ 48.192 ± 1.323
Photometric Color Index (PCI)	1.53 ± 0.12	2.64 ± 0.13	25
Peroxide Value	3.80 ± 0.44	13.00 ± 0.66	-
Free Acidity (%)	0.0487 ± 0.0143	0.1044 ± 0.0029	-
Acidity Index	0.0969 ± 0.0285	0.2078 ± 0.0058	-
Saponification Index	188.87 ± 1.79	186.86 ± 1.98	-

^a^ The average temperature at which γ was determined for Palm Oil. ^b^ The average temperature at which γ was determined for Recycled Palm Oil.

**Table 2:** The values of viscosity at different shear rates at 25 °C.

**Sample name**	**Shear rate (s^−1^)**			
	0.1	1	10	100
	**Viscosity (Pa·s)**			
R10	7010	678	54.5	4.31
EM-R10	6450	490	27.3	2.52

**Table 3:** Static contact angle (θ), adhesion (mechanical) work (W_a_), and solid–liquid interfacial tension (γ_sl_) for water.

Sample	Drop Image	θ (°)	W_a_ (mN/m)	γ_sl_ (mN/m)
R10	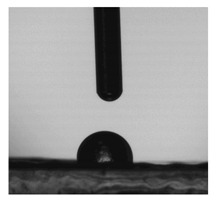	95 ± 5.7	66	69
EM-R10	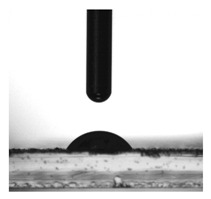	54 ± 0.98	116	9

**Table 4:** Static contact angle (θ), adhesion work (W_a_), and solid–liquid interfacial tension (γ_sl_) for ethylene glycol.

Sample	Drop Image	θ (°)	W_a_ (mN/m)	γ_sl_ (mN/m)
R10	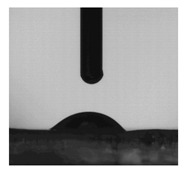	47 ± 4.59	80	30
EM-R10	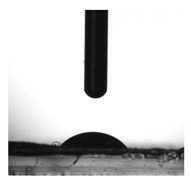	49 ± 1.20	79	21

**Table 5:** Melting peaks T_peak_ (°C).

Peak	UP Initial	UP Recycled	EM-UP Recycled	R10	EM-R10
1	-	−30.01	-	−38.42	-
2	−17.02	−17.43	−17.10	−24.84	−20.93
3	-	−13.10	-	-	-
4	−4.71	−4.86	−4.78	−6.93	-
5	2.20	2.30	2.63	2.48	0.48
6	4.52	5.05	-	-	-
7	5.78	-	5.63	-	5.82
8	11.22	8.72	9.14	10.40	-
9	16.63	15.06	-	-	-
10	23.47	23.32	20.81	20.89	24.90
11	30.45	28.31	-	-	-
12	-	34.73	36.73	36.82	-
13	-	43.48	-	-	41.57

**Table 6:** Melting enthalpy ΔH (J/g).

Peak	UP Initial	UP Recycled	EM-UP Recycled	R10	EM-R10
1	-	−0.033	-	−0.36	-
2	−0.32	−0.13	−0.68	−2.71	−2.91
3	-	−0.028	-	-	-
4	−0.32	−0.26	−0.43	−0.20	-
5	−3.76	−2.70	−5.93	−4.16	−3.54
6	−0.36	−1.40	-	-	-
7	−0.36	-	−0.67	-	−0.30
8	−0.47	−1.48	−1.90	−1.58	-
9	−0.84	−1.47	-	-	-
10	−0.15	−0.016	−10.24	−12.62	−1.84
11	−4.16	−3.64	-	-	-
12	-	−0.021	−1.52	−2.19	-
13	-	−6.18	-	-	−2.79

**Table 7:** Crystallization peaks T_peak_ (°C).

Peak	UP Initial	UP Recycled	EM-UP Recycled	R10	EM-R10
1	−56.16	−58.25	−57.83	−55.08	-
2	−47.32	−48.16	−51.24	-	-
3	−25.82	−20.66	−20.40	−20.82	−17.57
4	−5.63	−6.48	−6.22	−1.39	-
5	2.72	3.12	3.81	-	0.61
6	-	8.93	10.52	6.52	-
7	-	13.18	-	14.35	-
8	-	18.02	18.36	-	-
9	-	21.19	27.85	20.88	26.35
10	-	-	-	-	47.27

**Table 8:** Crystallization enthalpy ΔH (J/g).

Peak	UP Initial	UP Recycled	EM-UP Recycled	R10	EM-R10
1	0.56	0.25	0.53	2.75	-
2	0.26	0.44	0.061	-	-
3	0.55	0.11	0.16	0.18	0.15
4	0.51	0.42	0.49	4.69	-
5	14.96	14.37	18.77	-	6.40
6	-	0.030	0.023	1.12	-
7	-	0.15	-	0.097	-
8	-	1.22	4.98	-	-
9	-	0.61	0.055	5.69	5.71
10	-	-	-	-	3.31

**Table 9:** Antibacterial and Antifungal Activities of the Tested Compounds (mean ± SD, *n* = 3).

Compounds	Diameter of Inhibition Zones (mm)			
	*S. aureus* ATCC 25923	*E. coli* ATCC 25922	*Pseudomonas aeruginosa* ATCC 27853	*C. albicans* ATCC 10231
R10	20.0 ± 0.00	0	0	13.1 ± 0.05
EM-R10	11.1 ± 0.05	0	0	12.7 ± 0.06
M (DMSO)	0	0	0	0
Ciprofloxacin (5 µg/disk)	28.7 ± 0.06	30.0 ± 0.00	29.3 ± 0.57	NT *
Fluconazol (25 µg/disk)	NT *	NT *	NT *	22.0 ± 0.00

* NT—not tested.

## Data Availability

The original contributions presented in this study are included in the article. Further inquiries can be directed to the corresponding authors.

## Appendix A

**Table A1:** Components of the R10 Formulation Containing Recycled Palm Oil.

Phase	Raw Material Name	Content (%, *w*/*w*)
Phase A	Recycled palm oil	23.08
Phase A	Non-GMO soybean oil	23.08
Phase A	Shea butter (*Vitellaria paradoxa*)	9.62
Phase A	Olive-based emulsifying wax (sorbitan olivate)	9.62
Phase B	Apricot extract	13.46
Phase B	Pineapple extract	13.46
Phase C	Lime essential oil (*Citrus aurantifolia* oil)	0.96
Phase C	Fragard/Naticide	0.96
Phase C	Niacinamide	1.92
Phase C	Panthenol	3.85

**Table A2:** Components of the EM-R10 Formulation Containing Fir Bud Extract and Recycled Palm Oil.

Phase	Raw Material Name	Content (%, *w*/*w*)
Phase A	Fir bud extract in recycled palm oil	26.67
Phase A	Non-GMO soybean oil	26.67
Phase A	Shea butter (*Vitellaria paradoxa*)	11.11
Phase A	Olive-based emulsifying wax (sorbitan olivate)	11.11
Phase B	Organic green tea extract	15.56
Phase C	Lime essential oil (*Citrus aurantifolia* oil)	1.11
Phase C	Fragard/Naticide	1.11
Phase C	Niacinamide	2.22
Phase C	Panthenol	4.44

**Table A3:** Rheological Parameters of the Viscosity Curves Corresponding to the Herschel–Bulkley Model.

Sample Name	25 °C				35 °C			
	a	b	p	R^2^	a	b	p	R^2^
R10	6781.3	6097.9	0.01	0.9653	5796.2	5297.5	0.01	0.9871
EM-R10	5306.9	4888.3	0.01	0.8169	2077.3	1929.1	0.01	0.8680
